# A Case of Pigmented Paravenous Retinochoroidal Atrophy With Retinitis Pigmentosa

**DOI:** 10.7759/cureus.48532

**Published:** 2023-11-08

**Authors:** Atsuki Fukushima, Hitoshi Tabuchi

**Affiliations:** 1 Ophthalmology, Tsukazaki Hospital, Himeji, JPN; 2 Ophthalmology, Hiroshima University, Hiroshima, JPN

**Keywords:** visual field, retinitis pigmentosa, retinal dystrophy, pigmented paravenous retinochoroidal atrophy, fundus photograph

## Abstract

Chorioretinal atrophy with pigmentation along the retinal veins was observed in the right fundus of a 49-year-old patient. Extensive retinitis pigmentosa (RP) was observed in the left eye. Dynamic quantitative visual field testing revealed a scotoma in the right eye that corresponded to the area of ​​retinochoroidal atrophy and afferent visual field constriction was observed on the left eye. An electroretinogram test revealed that the right eye showed attenuated type and the left eye showed negative type. Thus, the conditions of his right eye and left eye were diagnosed as pigmented paravenous retinochoroidal atrophy (PPRCA) and RP, respectively. Thus, there may be a higher proportion of PPRCA patients with unilateral RP than expected.

## Introduction

Pigmented paravenous retinochoroidal atrophy (PPRCA) is a disease characterized by choroidal atrophy with bilateral contrasting bone-like pigmentation along the retinal veins [1]. This disease is classified mainly based on ophthalmoscopic findings and is initiated by many factors such as genetic disorders [2] and inflammation [3].

When considering genetic factors, a relationship with retinitis pigmentosa (RP) has been suspected due to the presence of bone-like pigmentation. We report a case of PPRCA in one eye and RP in the other eye.

## Case presentation

A 49-year-old male had been aware of visual impairment in his left eye for over 20 years, but he ignored it. He visited our hospital on March 3, 2020 with the chief complaint of visual disturbance of his right eye since last year. No notable abnormalities were found in the family history.

**Figure 1:**
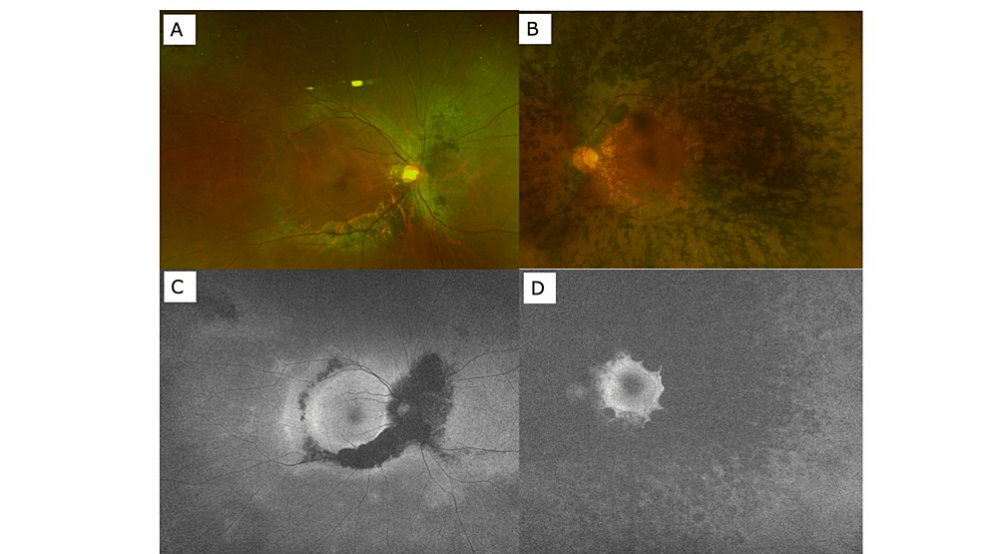
Wide-view fundus photograph (A: right eye, B: left eye) and autofluorescence fundus photograph (C: right eye, D: left eye) at the initial visit.

Corrected visual acuity at the first visit was 20/12.5 on the right and 20/25 on the left. Intraocular pressures were 16 mmHg on the right and 14 mmHg on the left. The cornea, anterior chamber, and lens were clear. Chorioretinal atrophy with pigmentation along the retinal veins was observed in the right fundus (Figure 1A). Autofluorescence was lost just in accord with the pigmented area (Figure 1C). Extensive RP was observed in the left eye as shown in the fundus photograph (Figure 1B) and the autofluorescence fundus photograph (Figure 1D). 

**Figure 2:**
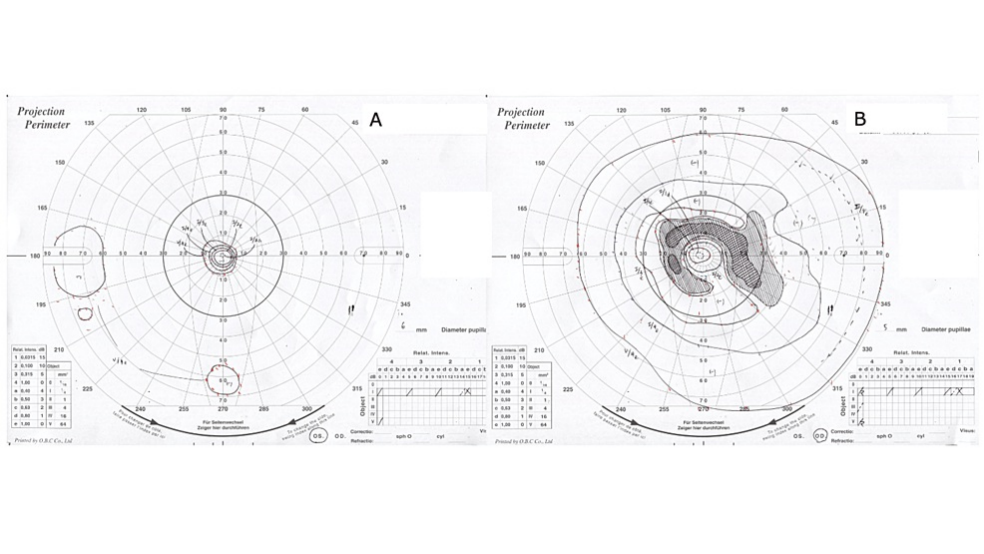
Dynamic quantitative visual field test (A: right eye, B: left eye) at the initial visit.

Dynamic quantitative visual field testing revealed a scotoma in the right eye that corresponded to the area of ​​retinochoroidal atrophy (Figure 2A). Afferent visual field constriction was observed in the left eye (Figure 2B).

**Figure 3:**
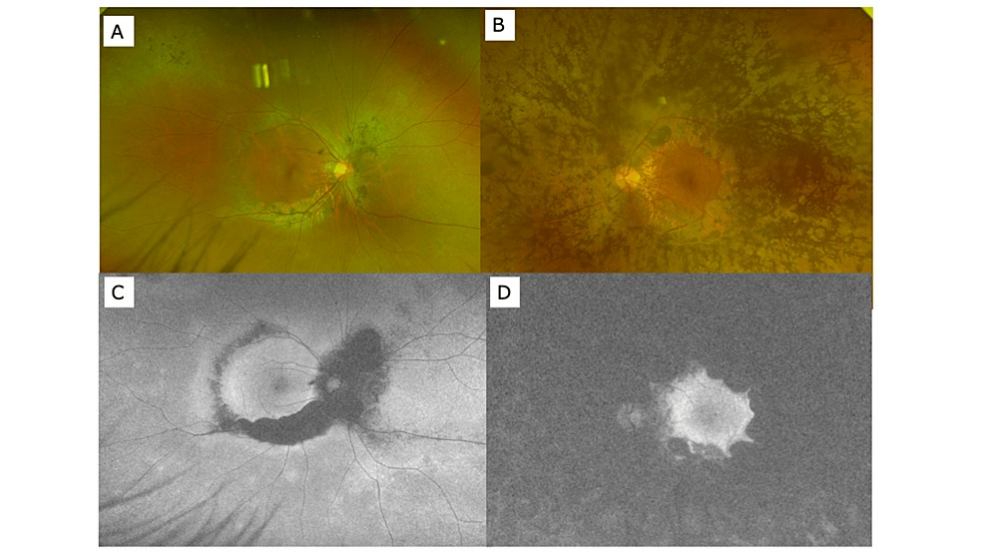
Wide-view fundus photograph (A: right eye, B: left eye) and autofluorescence fundus photograph (C: right eye, D: left eye) three years after the initial visit.

Since then, the patient has been diagnosed with RP. An electroretinogram test was performed on August 18, 2021, and the waveforms of right and left eye showed an attenuated and negative pattern, respectively (data not shown). Since then, regular follow-up has been performed, but no deterioration of the fundus (Figures 3A-3D) and visual field (data not shown) have been observed up to the three-year period.

**Figure 4:**
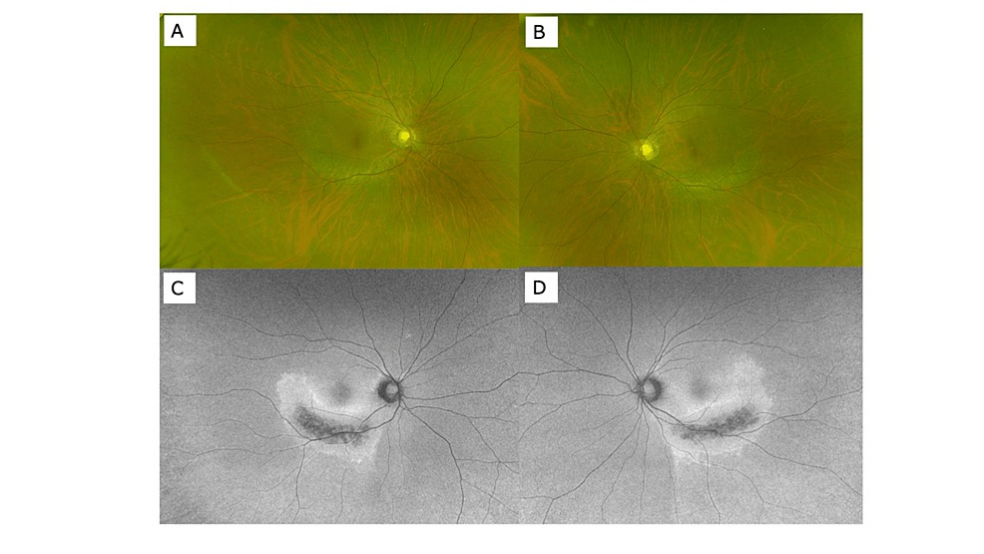
Wide-view fundus photograph (A: right eye, B: left eye) and autofluorescence fundus photograph (C: right eye, D: left eye) of a case of bilateral PPRCA (48-year-old female). PPRCA: Pigmented paravenous retinochoroidal atrophy

**Figure 5:**
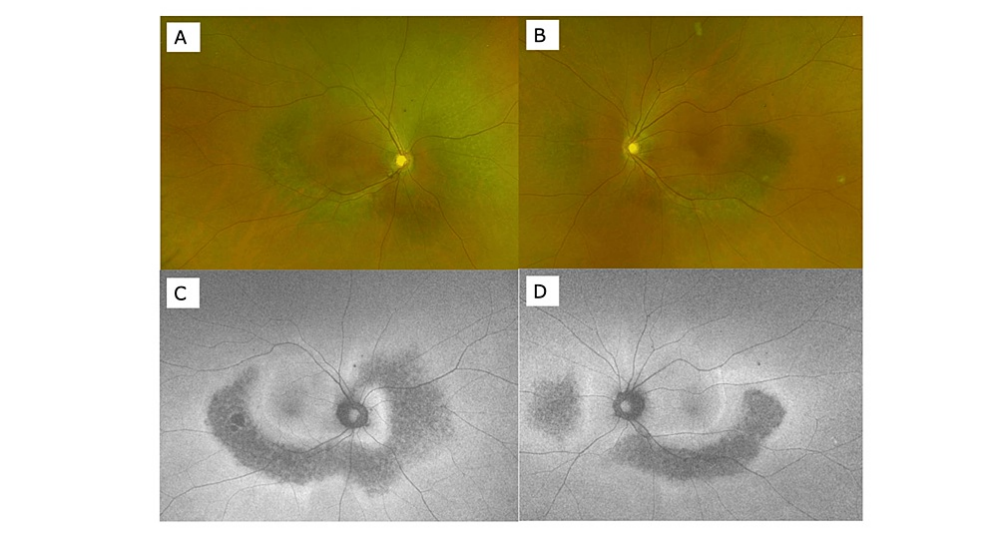
Wide-view fundus photograph (A: right eye, B: left eye) and autofluorescence fundus photograph (C: right eye, D: left eye) of a case of bilateral PPRCA (68-year-old female). PPRCA: Pigmented paravenous retinochoroidal atrophy

**Figure 6:**
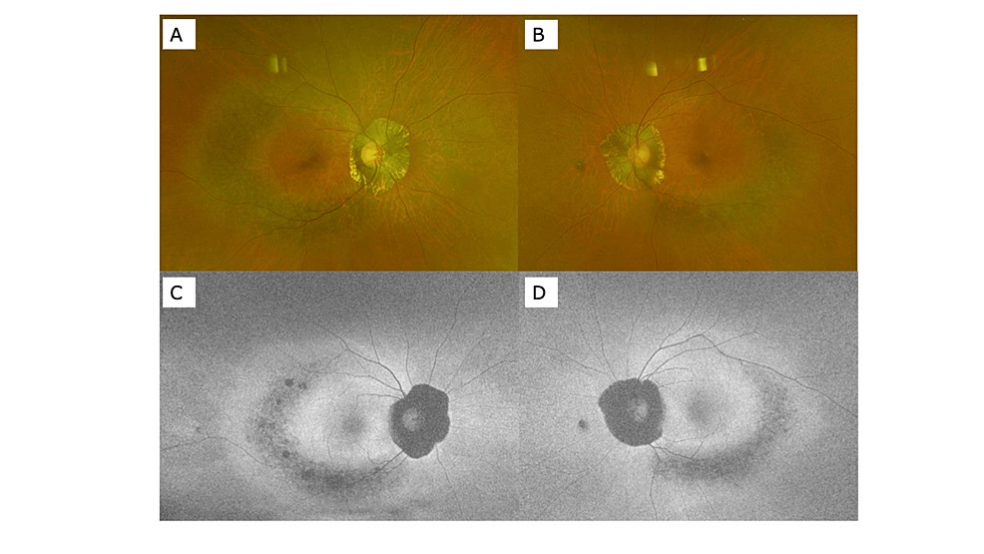
Wide-view fundus photograph (A: right eye, B: left eye) and autofluorescence fundus photograph (C: right eye, D: left eye) of a case of bilateral PPRCA (69-year-old female). PPRCA: Pigmented paravenous retinochoroidal atrophy

**Figure 7:**
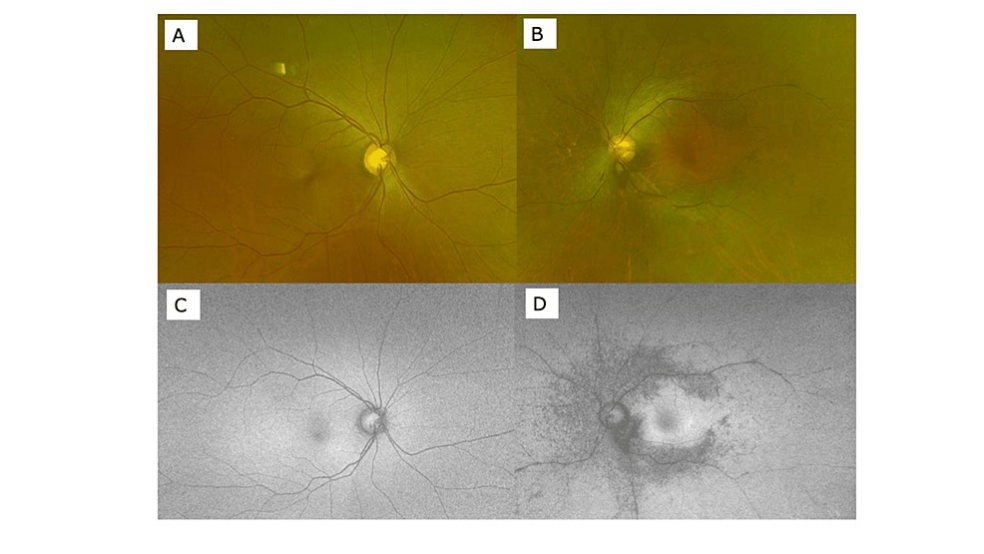
Wide-view fundus photograph (A: right eye, B: left eye) and autofluorescence fundus photograph (C: right eye, D: left eye) of a case of PPRCA in the left eye (69-year-old female). PPRCA: Pigmented paravenous retinochoroidal atrophy

In order to investigate the frequency of PPRCA patients with unilateral RP, we present fundus photographs of PPRCA cases we have experienced in the past three years. There were three cases of bilateral PPRCA (Figures 4-6), and one case where PPRCA was found in one eye, but no abnormality was found in the other eye (Figure 7).

## Discussion

In principle, both PPCRA [1,2] and RP are observed bilaterally and almost symmetrically. There is some overlap in findings between these two disease types. In fact, PPCRA is considered to be a precursor condition of RP, and there is also a report that PPRCA may be considered as a self-limited form of RP [4]. 

Indeed, genetic studies have shown the CRB1 (Crumbs 1) gene, the mutations within which are associated with both PPRCA and several types of retinal dystrophies, including RP 12, a severe type of RP [5]. More recently, RPGRIP1, a gene that has been associated with Leber congenital amaurosis [6] and cone-rod dystrophy [7], was identified to be pathogenic for PPCRA [8]. Thus, it is clear that RP and PPRCA are closely related from the viewpoint of genetic abnormalities.

There are several reports of PPCRA in one eye and RP in the other eye. Ratra et al., for the first time, reported concurrent manifestation of RP in one eye and PPRCA in the other eye found in a single patient [9]. Subsequently, Aoki et al. [5], Mehrotra et al. [10], and Kumar et al. [4] also reported similar cases. Among them, Kumar et al. reported that 2 out of 5 cases had PPRCA in one eye and RP in the other eye [4]. We experienced five cases of PPRCA from April 2021 to October 2023. The case presented here is one of these five cases, thus suggesting that 20% of cases with PPRCA manifest RP in the other eye. Although it has been previously recognized as rare, there may be a higher proportion of PPRCA patients with unilateral RP than expected.

In general, patients often develop RP or PPRCA in both eyes, and from the perspective of genetic abnormalities, it makes sense to find the same changes in both eyes. The mechanism by which RP develops in one eye and PPRCA develops in the other is unknown. The mechanism will become clearer as research into the relationship between genes and phenotypes progresses.

## Conclusions

PPRCA is considered to be bilateral and almost symmetrical. However, reports of cases with PPRCA in one eye with RP in the other eye are increasing, due to the fact of genetical close linkage between PPRCA and RP. As we reported here, PPRCA patients with unilateral RP may not be so rare.

## References

[1] Huang HB, Zhang YX (2014). Pigmented paravenous retinochoroidal atrophy (review). Exp Ther Med.

[2] Antropoli A, Arrigo A, Pili L (2023). Pigmented paravenous chorioretinal atrophy: updated scenario. Eur J Ophthalmol.

[3] Ramtohul P, Comet A, Gascon P, Denis D (2020). Pigmented paravenous retinochoroidal atrophy associated with Vogt-Koyanagi-Harada disease: a case report. BMC Ophthalmol.

[4] Kumar V, Kumawat D, Tewari R, Venkatesh P (2019). Ultra-wide field imaging of pigmented para-venous retino-choroidal atrophy. Eur J Ophthalmol.

[5] Aoki S, Inoue T, Kusakabe M (2017). Unilateral pigmented paravenous retinochoroidal atrophy with retinitis pigmentosa in the contralateral eye: a case report. Am J Ophthalmol Case Rep.

[6] Khan AO, Al-Mesfer S, Al-Turkmani S, Bergmann C, Bolz HJ (2014). Genetic analysis of strictly defined Leber congenital amaurosis with (and without) neurodevelopmental delay. Br J Ophthalmol.

[7] Hameed A, Abid A, Aziz A, Ismail M, Mehdi SQ, Khaliq S (2003). Evidence of RPGRIP1 gene mutations associated with recessive cone-rod dystrophy. J Med Genet.

[8] Bianco L, Antropoli A, Arrigo A (2023). RPGRIP1 variant associated with pigmented paravenous chorioretinal atrophy. Eur J Ophthalmol.

[9] Ratra D, Chandrasekharan DP, Aruldas P, Ratra V (2016). Concurrent retinitis pigmentosa and pigmented paravenous retinochoroidal atrophy phenotypes in the same patient. Indian J Ophthalmol.

[10] Mehrotra N, Khandelwal J, Nagpal M (2019). Rare case of simultaneous manifestation of pigmented paravenous retinochoroidal atrophy and retinitis pigmentosa in contralateral eye. Indian J Ophthalmol.

